# Additions to Bambusicolous Fungi of Savoryellaceae from Southwest China

**DOI:** 10.3390/jof9050571

**Published:** 2023-05-14

**Authors:** Xian-Dong Yu, Sheng-Nan Zhang, Jian-Kui Liu

**Affiliations:** Center for Informational Biology, School of Life Science and Technology, University of Electronic Science and Technology of China, Chengdu 611731, China

**Keywords:** asexual and sexual morphs, bamboo fungi, freshwater fungi, phylogeny, taxonomy, three new species

## Abstract

Asexual and sexual morphs of saprobic bambusicolous fungi were collected from freshwater and terrestrial habitats in Sichuan Province, China. Taxonomic identification of these fungi was carried out on the basis of morphological comparison, culture characteristics, and molecular phylogeny. Multi-gene phylogeny based on combined SSU, ITS, LSU, *rpb2,* and *tef1α* sequence data was performed to determine their phylogenetic placement, and the result showed that these fungi belong to Savoryellaceae. Morphologically, four asexual morphs are similar to *Canalisporium* and *Dematiosporium*, while a sexual morph well-fits to *Savoryella*. Three new species, *Canalisporium sichuanense*, *Dematiosporium bambusicola*, and *Savoryella bambusicola* are identified and described. Two new records, *C. dehongense* and *D. aquaticum,* were recovered from the bamboo hosts in terrestrial and freshwater habitats, respectively. In addition, the nomenclatural confusion of *C. dehongense* and *C. thailandense* is discussed.

## 1. Introduction

Bamboo fungi are a highly diverse group of organisms [1,2,3,4,5], and research on bambusicolous fungi also provides opportunities to control bamboo diseases and promote bamboo cultivation [6]. Approximately 150 basidiomycetes and 1150 ascomycetes have been reported from bamboo, including 350 asexual morphs, 240 hyphomycetes, and 110 coelomycetes [4,6]. China is rich in bamboo resources, and its bamboo species account for more than 50% of the world’s bamboo [7,8]. Sichuan Province has a variety of bamboo forests and inhabits a large number of bamboo fungi due to its complex topography and climate conditions [9]. Some well-represented families of bamboo fungi in Sichuan Province are Astrosphaeriellaceae [10], Bambusicolaceae [9,11], Occultibambusaceae [9,12] distributed in Dothideomycetes, and Apiosporaceae [12,13], Nectriaceae [10,14], Phyllachoraceae [10,15] distributed in Sordariomycetes.

Savoryellaceae (Savoryellales, Sordariomycetes), with an estimated divergence crown age of 182 MYA [16], contains generally saprobic fungi on bamboos, palms, *Pandanus*, *Machilus* sp., *Pinus* sp., or other unidentified woody substrates in terrestrial, freshwater, marine, brackish water, and water-cooling towers [16,17,18,19,20,21,22]. The family currently comprises six genera: *Ascotaiwania*, *Bactrodesmium, Canalisporium*, *Dematiosporium, Neoascotaiwania,* and *Savoryella* [17,23], which are characterized by immersed or superficial, globose to pyriform ascomata with paraphyses, two-to-eight-spored, clavate to cylindrical, unitunicate asci with an inamyloid apical ring, and ellipsoid, fusiform ascospores, with or without a gelatinous sheath, as well as a dematiaceous hyphomycetous asexual morph with globose to subglobose or obovate to oval conidia [24,25].

Members of Savoryellaceae are often found in freshwater habitats [17,23,26]. For example, the genus *Canalisporium* is found in freshwater habitats and typified by *C. caribense* [27]. Most *Canalisporium* species are characterized by dark brown and muriform conidia [27]; *Canalisporium grenadoideum* (=*Ascothailandia grenadoidea*) is the only species that represents a sexual morph: globose, dark brown, ostiolate ascomata, eight-spored, long cylindrical, unitunicate asci, fusiform, three-euseptate ascospores [28]. *Dematiosporium*, typified by *D. aquaticum*, and currently consist of species, in particular, from freshwater habitat [17,26]. This genus is characterized by cylindrical, unbranched, aseptate conidiophores and globose to subglobose, dictyospores conidia with a pore in each cell [17,26]. The generic type of *Savoryella*, *S. lignicola*, was initially discovered during a survey of cellulolytic fungi in a simulated aquatic environment and has been subsequently recovered from different woody substrates (i.e., *Bambusa* sp.) in aquatic or marine habitats [18,21,29]. Members of *Savoryella* are characterized by immersed, partly immersed, or superficial, globose, subglobose, or ellipsoidal ascostromata, two-to-eight-spored, cylindrical or clavate, unitunicate asci with an apical truncate non-amyloid apical thickening containing a pore and uni- or biseriate, ellipsoidal, three-septate ascospores [18,30]; as well as dematiaceous hyphomycetes, which produce micronematous conidiophores, holoblastic, terminal, and intercalary conidiogenous cells, solitary or aggregated, pyriform to obovoid, septate conidia [19].

During a survey of saprobic fungi from bamboo in Sichuan Province, China, a series of asexual and sexual fungi were collected. Multi-gene phylogeny integrated with morphological comparison was carried out to determine the taxonomic status of these new collections, of which new species and new host records contributed to *Canalisporium*, *Dematiosporium,* and *Savoryella* were introduced and justified to the Savoryellaceae.

## 2. Materials and Methods

### 2.1. Isolation and Morphological Examination

Fungi associated with decaying bamboo were collected from Dujiangyan and Qionglai in Sichuan Province, China in 2021. Specimens were placed in envelopes or plastic bags and taken to the laboratory. Morphological characteristics were observed using a Nikon ECLIPSE E200 stereo microscope and photographed by a Nikon ECLIPSE Ni-U compound microscope fitted with a Nikon DS-Ri2 digital camera as per the guidelines provided in Senanayake et al. [31]. Single-spore isolations were used to obtain pure cultures following the methods described by Senanayake et al. [31]. Measurements were made with the Tarosoft (R) Image Framework program v. 0.9.7, following Liu et al. [32]. Photo plates representing fungal structures were processed in Adobe Photoshop CS6 software (Adobe Systems Inc., San Jose, CA, USA). Herbarium specimens (dry branches with fungal material) were deposited in the herbarium of Cryptogams, Kunming Institute of Botany Academia Sinica (KUN-HKAS), Kunming, China and the herbarium of the University of Electronic Science and Technology (HUEST), Chengdu, China. The isolates obtained in this study were deposited in China General Microbiological Culture Collection Center (CGMCC) in Beijing, China and the University of Electronic Science and Technology Culture Collection (UESTCC) in Chengdu, China. Names of the new taxa were registered in MycoBank [33].

### 2.2. DNA Extraction, PCR Amplification and Sequencing

A Trelief TM Plant Genomic DNA Kit (Beijing TsingKe Biotech Co., Ltd., Beijing, China) was used to extract total genomic DNA from fungal mycelia. DNA amplification was performed by a polymerase chain reaction (PCR). Five partial gene regions, the small subunit of nuclear ribosomal RNA gene (SSU), the nuclear ribosomal internal transcribed spacer (ITS: ITS1-5.8S-ITS2), the large subunit of nuclear ribosomal RNA gene (LSU), the second-largest subunit of RNA polymerase II (*rpb2*), and the translation elongation factor 1-alpha (*tef1α*), were used in this study. Sequences of SSU, ITS, LSU, *rpb2,* and *tef1α* were amplified using primer pairs NS1/NS4, ITS5/ITS4, LR0R/LR5, fRPB2-5F/fRPB2-7cR, and 983F/2218R, respectively [34,35,36,37]. The amplification reactions were performed in 50 μL PCR mixtures containing 25 μL 2× Taq Plus MasterMix (Dye) (CoWin Biosciences, (Taizhou), Co., Ltd., Taizhou, China), 20 μL ddH_2_O, 1μL DNA template, and 2μL of each primer (10µM/L). The PCR thermal cycle program for SSU, ITS, LSU, *rpb2,* and *tef1α* amplification was as follows: initial denaturing step of 94 °C for 5 min, followed by 35 cycles of denaturation at 94 °C for 30 s, annealing at 56 °C (SSU, ITS, LSU, *rpb2*), 62 °C (*tef1α*) for 30 s, elongation at 72 °C for 30 s, and final extension at 72 °C for 5 min. PCR products were checked on 1% agarose electrophoresis gels stained with Gel Red. The sequencing reactions were carried out with primers mentioned above by Beijing Tsingke Biotechnology Co., Ltd., Chengdu, China.

### 2.3. Phylogenetic Analyses

The BLAST searches were performed for finding similar sequences that match our data. A concatenated dataset of the SSU, ITS, LSU, *rpb2,* and *tef1α* sequences were used for phylogenetic analyses with the inclusion of reference taxa from GenBank (Table 1). The sequences were aligned by using the online multiple alignment program MAFFT v.7 (http://mafft.cbrc.jp/alignment/server/ (accessed on 27 May 2022)) [38], and the alignment was manually optimized in BioEdit v.7.0.9 [39]. The five-gene dataset was concatenated by Mesquite v. 3.11 (http://www.mesquiteproject.org/ (accessed on 27 May 2022)) for multi-gene phylogenetic analyses. Maximum likelihood (ML) and Bayesian inference (BI) were carried out as detailed in Dissanayake et al. [40]. Maximum likelihood (ML) analysis was performed using RAxML-HPC v.8 tool via the CIPRES Science Gateway V3.3 (https://www.phylo.org/portal2/home.action, accessed on 27 May 2022) with rapid bootstrap analysis, and a general time-reversible model (GTR) was applied with a discrete GAMMA distribution. The Bayesian inference (BI) analyses were performed by using PAUP v.4.0b10 [41] and MrBayes v. 3.1.2 [42,43]. The best model for different genes partition in the concatenated dataset was determined by MrModeltest 2.3. [44], and posterior probabilities were determined by Markov Chain Monte Carlo sampling (MCMC) in MrBayes v.3.1.2 [42,43]. The final alignment and phylogram were submitted to TreeBASE (https://www.treebase.org/, accessed on 5 May 2023, submission ID: 30350). The phylogenetic trees were visualized by Treeview v. 1.6.6 [45].

## 3. Results

### 3.1. Phylogenetic Analyses

**Figure 1:**
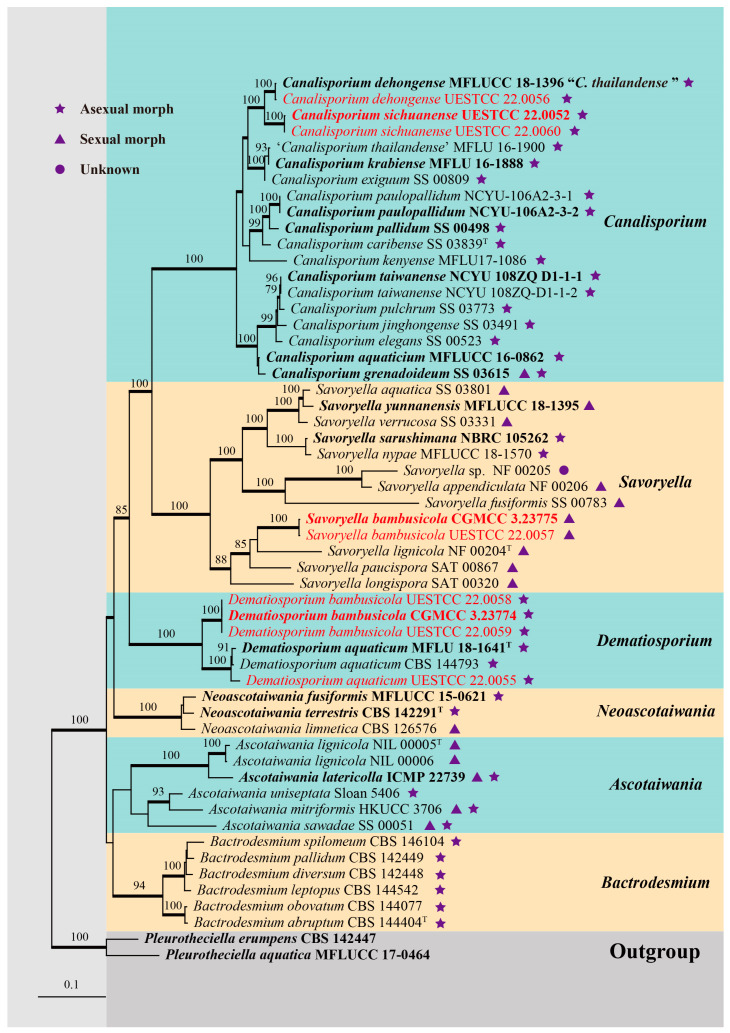
RAxML tree generated from combined SSU, ITS, LSU, *rpb2,* and *tef1α* sequence data of Savoryellaceae. Bootstrap values for ML equal to or greater than 75% are placed above the branches. Branches with Bayesian posterior probabilities (BYPP) from MCMC analysis equal to or greater than 0.95 were in bold. The ex-type strains were indicated in bold, and newly generated sequences were indicated in red. “T” represents the type species of each genus.

To determine the phylogenetic placement of the new collections in this study, the combined SSU, ITS, LSU, *rpb2,* and *tef1α* data set comprised 55 sequences with *Pleurotheciella aquatica* (MFLUCC 17-0464) and *P. erumpens* (CBS 142447) as the outgroup taxa. The concatenated matrix comprised a total of 4870 characters (SSU: 1081 bp; ITS: 841 bp; LSU: 921 bp; *rpb2*: 1056 bp; *tef1α*: 971 bp) including gaps. Maximum likelihood (ML) and Bayesian inference (BI) analyses were resulting in generally congruent topologies. The best-scoring ML tree (Figure 1) was selected to represent the relationships among taxa, in which a final likelihood value of –37,950.559206 is presented. The evolutionary models for Bayesian analysis were selected for each locus, and the best-fit model GTR+I+G for ITS, LSU, *rpb2,* and *tef1α*, SYM+I+G for SSU, respectively. Six simultaneous Markov chains were run for 165,000 generations, and trees were sampled every 1000 generations and 165 trees were obtained. The first 33 trees representing the burn-in phase of the analyses were discarded, while the remaining 132 trees were used for calculating posterior probabilities in the majority rule consensus tree (critical value for the topological convergence diagnostic is 0.01).

Forty-three representative species of Savoryellaceae are included in our phylogenetic analysis (Figure 1). Two isolates of *Canalisporium sichuanense* (CGMCC 3.23926, UESTCC 22.0060) were sister to *C. dehongense* (MFLUCC 18-1396 and UESTCC 22.0056) and were well supported (100% ML/1.00 BYPP). Three isolates of *Dematiosporium bambusicola* (CGMCC 3.23774, UESTCC 22.0058, UESTCC 22.0059) formed a distinct clade sister to *Dematiosporium aquaticum* (CBS 144793, MFLU 18-1641, UESTCC 22.0055) with high statistical support (100% ML/1.00 BYPP). Two isolates of *Savoryella bambusicola* (CGMCC 3.23775 and UESTCC 22.0057) were nested in the genus *Savoryella* and close to *S. lignicola* (NF 00204).

### 3.2. Taxonomy

***Canalisporium dehongense*** W. Dong, H. Zhang, and K.D. Hyde, Fungal Diversity 96: 159 (2019) Figure 2 and Figure 3

*MycoBank*: MB 555407

**Figure 2:**
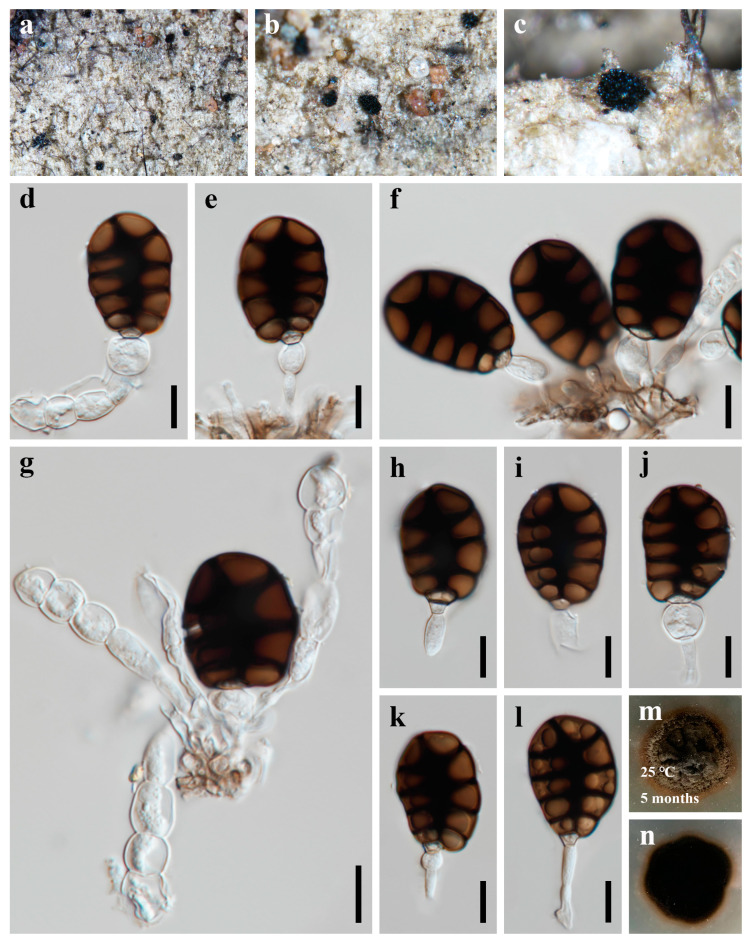
***Canalisporium dehongense*** (HUEST 22.0057) (**a**–**c**) colonies on bamboo substrate. (**d**–**g**) Conidiophores and conidia. (**h**–**l**) Conidia with conidiogenous cells. (**m**,**n**) Colonies on PDA, above (**m**) and below (**n**). Scale bars: (**d**–**l**) = 10 μm.

**Figure 3:**
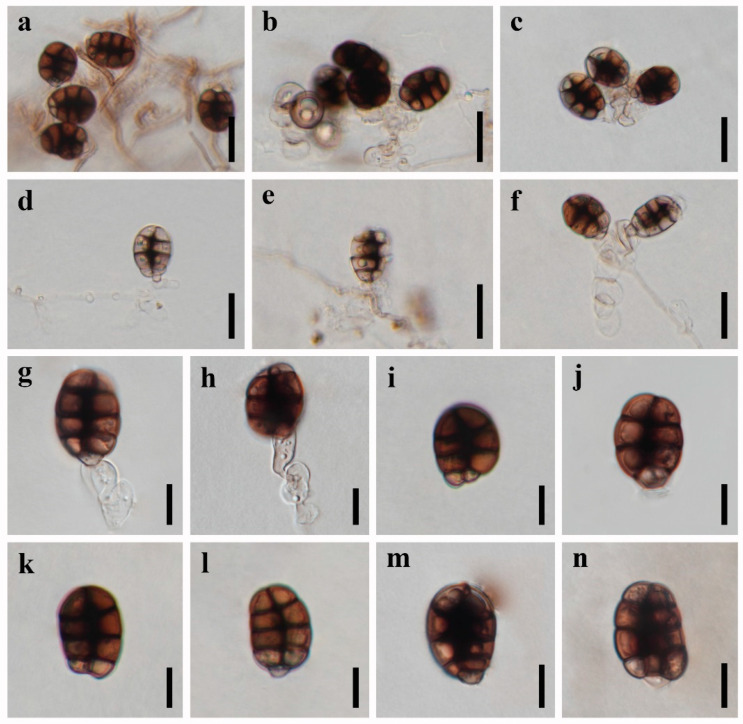
**Asexual reproduction of *Canalisporium dehongense*** (UESTCC 22.0056) on PDA medium about three months. (**a**) Hyphae and conidiophores with conidia. (**b**–**h**) Conidiogenous cells and conidia. (**i**–**n**) Conidia. Scale bars: (**a**–**f**) = 20 μm, (**g**–**n**) = 10 μm.

*Saprobic* on dead branches of bamboo in terrestrial habitat. **Sexual morph**: Undetermined. **Asexual morph**: hyphomycetous (Figure 2 and Figure 3). *Colonies* sporodochial, scattered, punctiform, pulvinate, granular, black, glistening. *Mycelium* immersed, consisting of branched, septate, thin-walled, smooth, pale to brown hyphae. *Conidiophores* up to 60 μm long, micronematous, mononematous, vesiculate, consisted of one to five subglobose to cylindrical, hyaline cells, smooth, unbranched, septate, constricted at the septa. *Conidiogenous cells* 8.5–12.5 × 7–10 µm ($\overset{¯}{x}$ = 9.8 × 8.8 µm, n = 10), holoblastic, monoblastic, integrated, terminal, determinate, subglobose, ellipsoidal, sometimes cuneiform, hyaline, smooth, thin-walled. *Conidia* 25–32 × 17–23 µm ($\overset{¯}{x}$ = 29 × 20 µm, n = 50), solitary, acrogenous, ellipsoidal to obovoid, muriform, smooth, brown, comprising of one straight column of vertical septa and three to four rows of transverse septa, slightly constricted at the septa, darkened and thickly banded at the septa, canals in the septa obscured by dark pigmentation; basal cell single, cuneiform, 4.5–6.5 µm wide, sometimes swollen, hyaline to pale brown.

*Culture characteristics*: Colonies on PDA reaching 20–30 mm after 5 months at 25 °C, circular, raised to umbonate, rough surface, dense, entire at the edge, brown, dry, reverse dark brown to black. *Mycelium* subhyaline to pale brown, 1.5–2.5 µm wide in culture. *Conidiophores* subhyaline. *Conidiogenous cells* integrated, subhyaline. *Conidia* pale brown to brown, septate, subglobose to obovoid, muriform, smooth, constricted at the septa, 20–25 µm ($\overset{¯}{x}$ = 22.5 µm, n = 30) long, 15–17 µm ($\overset{¯}{x}$ = 16.5 µm, n = 30) wide (Figure 3).

*Material examined*: CHINA, Sichuan Province, Chengdu City, Qionglai County, Chuanxi Bamboo Sea Area, 30°20′32″ N, 103°18′26″ E, 540 m elevation, on dead branches of bamboo in terrestrial habitat, 12 October 2021, X.D. Yu, A5 (HUEST 22.0057); living culture UESTCC 22.0056.

*Notes*: *Canalisporium dehongense* was introduced by Hyde et al. [46] from a submerged wood in Yunnan Province, China and has been considered as a later synonym of *C. thailandense* [47]. However, Goh and Kuo [48] treated *Canalisporium dehongense* and *C. thailandense* as distinct species, and this is followed in this study. The new collection clustered with *Canalisporium dehongense* in the single- and multi-gene phylogeny and share the similar conidiogenous cells and conidial morphology. Therefore, we identify our collection as *C. dehongense,* and an additional bamboo host record is provided herein.

**Figure 4:**
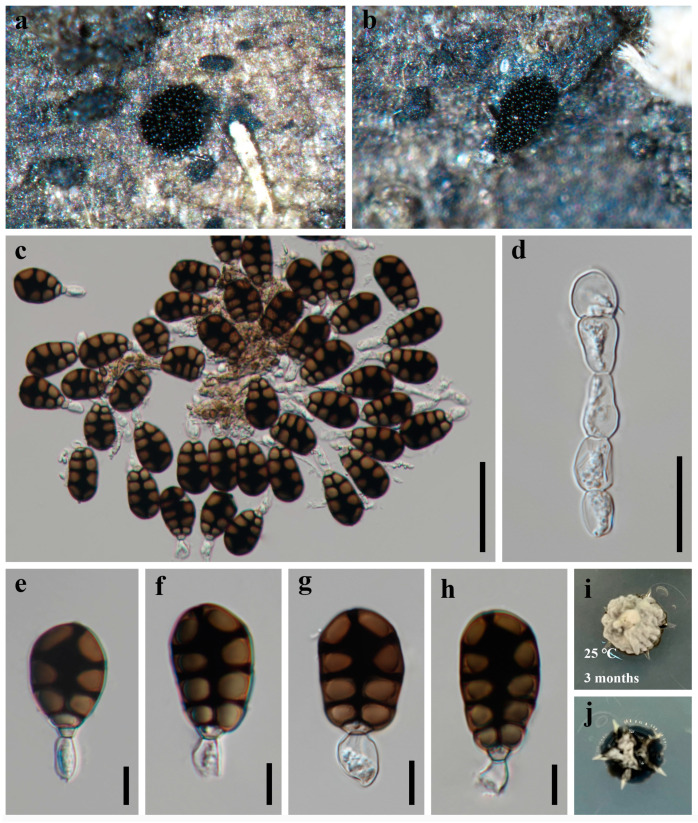
***Canalisporium sichuanense*** (HKAS 124625, **holotype**) (**a**–**h**) colonies on natural substrate. (**a**,**b**) Sporodochia on a bamboo substrate. (**c**) Squash mount of sporodochium showing conidiophores and conidia. (**d**) Conidiophores comprising of vesiculate cells. (**e**–**h**) Conidia with conidiogenous cells. (**i**,**j**) Colonies on PDA, above (**i**) and below (**j**). Scale bars: (**c**) = 50 μm, (**d**) = 20 μm, (**e**–**h**) = 10 μm.

**Figure 5:**
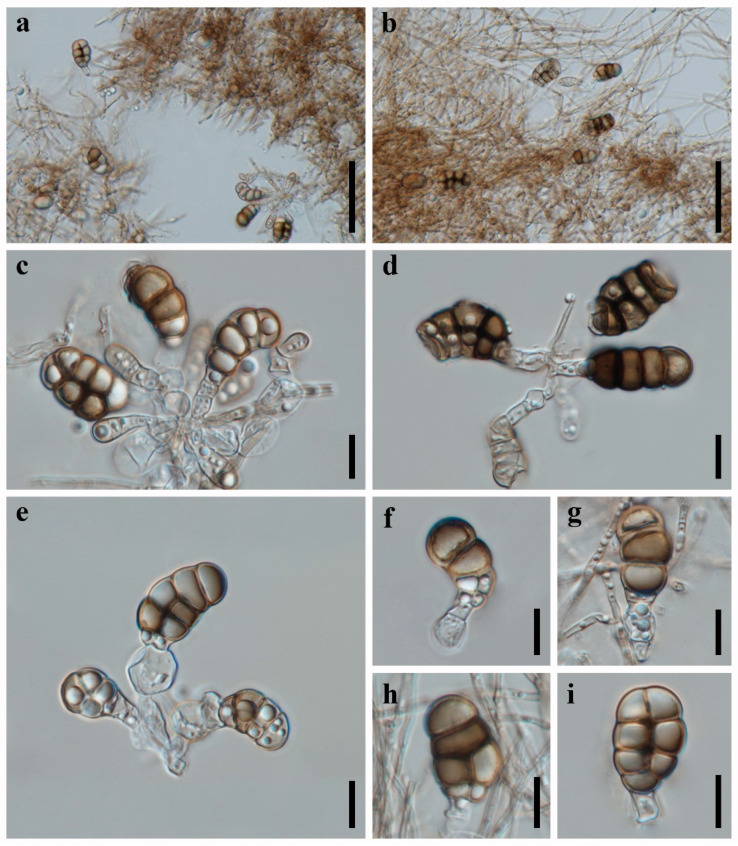
**Asexual reproduction of *Canalisporium sichuanense*** (CGMCC 3.23926, **ex-type**) on PDA medium about three months. (**a**,**b**) Hyphae and conidiophores with conidia. (**c**–**i**) Conidiogenous cells and conidia. Scale bars: (**a**,**b**) = 50 μm, (**c**–**i**) = 10 μm.

***Canalisporium sichuanense*** X.D. Yu, S.N. Zhang, and Jian K. Liu, sp. nov., Figure 4 and Figure 5

*MycoBank*: MB 847552

*Etymology*: The epithet refers to Sichuan Province where the fungus was collected.

*Holotype*: HKAS 124625

*Saprobic* on dead branches of bamboo in terrestrial habitat. **Sexual morph**: Undetermined. **Asexual morph**: hyphomycetous (Figure 4 and Figure 5). *Colonies* sporodochial, scattered, punctiform, pulvinate, granular, black, and shiny. *Mycelium* immersed, consisting of branched, septate, thin-walled, smooth, hyaline to pale brown hyphae. *Conidiophores* up to 60 µm long, micronematous, mononematous, vesiculate, consisted of one to six subglobose to cylindrical, hyaline cells, smooth, unbranched, septate, constricted at the septa. *Conidiogenous cells* 11–16 × 8–10 µm ($\overset{¯}{x}$ = 13.5 × 9 µm, n = 10), holoblastic, monoblastic, integrated, determinate, terminal, cylindrical, sometimes swelling to subglobose, hyaline, smooth, thin-walled. *Conidia* 27–37 × 17–21 µm ($\overset{¯}{x}$ = 31 × 20 µm, n = 50), solitary, acrogenous, cylindrical to obovoid, muriform, smooth, brown, comprising of a single, straight column of vertical septa and two to four rows of transverse septa, slightly constricted at the septa, darkened and thickly banded at the septa, canals in the septa obscured by dark pigmentation; basal cell cuneiform, 5.0–7.0 µm wide, sometimes swollen, hyaline to pale brown.

*Culture characteristics*: Colonies on PDA reaching 5–10 mm after 3 months at 25 °C, irregular, raised to umbonate, surface rough, dense, greyish-white, dry, reverse dark brown. *Mycelium* subhyaline to pale brown, 1.5–2.5 µm wide in culture. *Conidiophores* (Figure 5) subhyaline, 10–35 × 3.5–10.5 µm ($\overset{¯}{x}$ = 20.0 × 6.5 µm, n = 10). *Conidiogenous cells* (Figure 5) integrated, subhyaline, 4.5–10.5 × 5.5–10.5 µm ($\overset{¯}{x}$ = 8 × 7.5 µm, n = 10). *Conidia* (Figure 5) pale brown to brown, septate, cylindrical to obovoid, muriform, smooth, constricted at the septa, and the septa becoming progressively darker with conidial maturity, 15–24 µm ($\overset{¯}{x}$ = 20.5 µm, n = 30) long × 9–15 µm ($\overset{¯}{x}$ = 12.0 µm, n = 30) wide.

*Material examined*: CHINA, Sichuan Province, Chengdu City, Qionglai County, Lugou Bamboo Sea Area, 30°22′37″ N, 103°16′45″ E, 730 m elevation, on dead branches of bamboo in a terrestrial habitat, 12 October 2021, X.D. Yu, A44 (HKAS 124625, holotype); ex-type living culture CGMCC 3.23926; Dujiangyan County, Qingcheng Mountain, 30°54′29″ N, 103°32′36″ E, 960 m elevation, on dead branches of bamboo in a terrestrial habitat, 02 December 2021, Y. Yang, Q10-4 (HUEST 22.0061, paratype); ex-paratype living culture UESTCC 22.0060.

*Notes*: Morphologically, *Canalisporium sichuanense* resembles *C. dehongense* and *C. thailandense* in having hyaline, septate conidiophores, holoblastic, monoblastic conidiogenous cells, and muriform conidia [46,49]. However, they can be distinguished by the size of the basal cell (ca. 4 μm long in *C. sichuanense*, ca. 5 μm long in *C. dehongense*, ca. 2 μm long in *C. thailandense*) and conidial size (27–37 × 17–21 μm in *C. sichuanense*, 20–30 × 12–19 μm in *C. dehongense*, 22.5–31 × 17–22 μm in *C. thailandense*) [46,49]. Phylogenetically, they are distinct from each other (Figure 1), of which *C. sichuanense* is closer and sister to *C. dehongense* with high statistical support (100% ML/1.00 BYPP). The comparison of nucleotide differences between *C. sichuanense* and *C. dehongense* (MFLUCC 18-1396) showed that there are 0.10% (1/970) differences in SSU, 6.69% (44/658, including six gaps) in ITS, and 1.45% (11/761) in LSU gene regions, which also supports the introduction of *C. sichuanense* as a new species.

***Dematiosporium aquaticum*** Z.L. Luo, K.D. Hyde, and H.Y. Su, Fungal Diversity 99: 573 (2019) Figure 6

*MycoBank*: MB 555673

*Saprobic* on dead bamboo in freshwater habitat. **Sexual morph**: Undetermined. **Asexual morph**: hyphomycetous. *Colonies* on natural substratum, effuse, scattered to gregarious, dark brown to black, glistening, superficial. *Mycelium* immersed in natural substrate, unbranched, septate, hyaline to subhyaline hyphae. *Conidiophores* reduced to conidiogenous cells, or rarely 20–30 × 3.5–4.5 µm, micronematous, mononematous, cylindrical, unbranched. *Conidiogenous cells* not observed. *Conidia* 21–28 µm diam ($\overset{¯}{x}$ = 25 µm, n = 50), solitary, acrogenous, globose to subglobose, brown to dark brown, dictyospores, slightly constricted at the septa, with a pore in each cell.

*Culture characteristics*: Colonies on PDA reaching 10–15 mm after 7 months at 25 °C, circular, with dense mycelium on the surface, dark grayish of the inner ring, and brown.

*Material examined*: CHINA, Sichuan Province, Chengdu City, Qionglai County, Lugou Bamboo Sea Area, 30°22′37″ N, 103°16′45″ E, 730 m elevation, on dead branches of bamboo in a freshwater habitat, 12 October 2021, X.D. Yu, A1 (HUEST 22.0056); living culture UESTCC 22.0055.

*Notes*: Luo et al. [26] introduced a monotypic genus *Dematiosporium*, which was collected from decaying submerged wood in Erhai lake, Dali City, Yunnan Province, China. Subsequently, Réblová et al. [17] recollected and described it in France, where it occurs on decaying submerged wood of *Alnus glutinosa* and other unidentified substrates. Species of *Dematiosporium* are often found on decaying wood in aquatic environments and it is characterized by cylindrical, unbranched, aseptate conidiophores and globose to subglobose, dictyospores conidia with a pore in each cell [17,26]. Our new collection was found from decaying bamboo in freshwater environments. We identify it as *D. aquaticum* based on the morphological characters and phylogenetic analyses. The SSU, ITS, *rpb2,* and *tef1α* regions were attempted, and we were able to obtain the LSU sequences. This is the first report of *D. aquaticum* from bamboo in a freshwater habitat. We describe *D. aquaticum* in detail and supplement the description of the conidiophore that was missing from the previous description.

**Figure 7:**
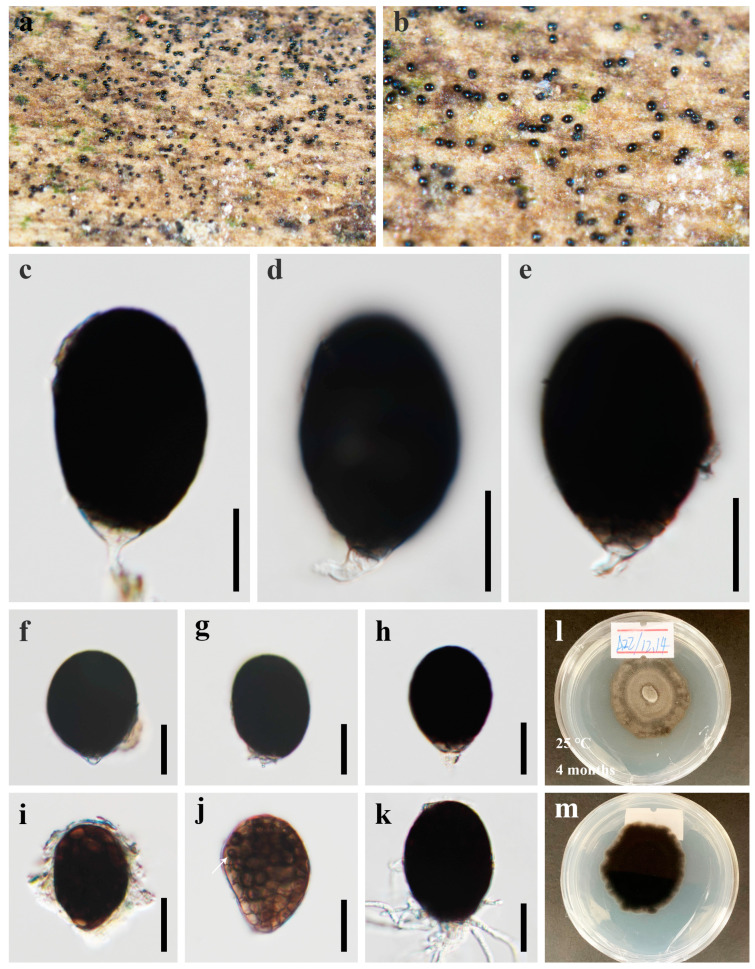
***Dematiosporium bambusicola*** (HKAS 124626, **holotype**) (**a**,**b**) colonies on a bamboo substrate. (**c**–**e**) Conidiophores and conidia. (**f**–**j**) Conidia. (**j**) The arrow indicated pore. (**k**) Germinating conidium. (**l**,**m**) Colonies on PDA, above (**l**) and below (**m**). Scale bars: (**c**–**k**) = 20 μm.

***Dematiosporium bambusicola*** X.D. Yu, S.N. Zhang, and Jian K. Liu, sp. nov., Figure 7.

*MycoBank*: MB 847553

*Etymology*: Refers to the bamboo host from which the fungi was found.

*Holotype*: HKAS 124626

*Saprobic* on dead bamboo in freshwater habitat. **Sexual morph**: Undetermined. **Asexual morph**: hyphomycetous. *Colonies* on natural substratum, effuse, scattered to gregarious, dark brown to black, glistening, superficial. *Mycelium* immersed in natural substrate, composed of hyaline, septate, branched, smooth hyphae. *Conidiophores* 6.5–11.5 × 2–4.5 µm ($\overset{¯}{x}$ = 9 × 3 µm, n = 10), micronematous, mononematous, cylindrical, straight or slightly flexuous, unbranched, aseptate. *Conidiogenous cells* holoblastic, monoblastic, cuneiform, integrated, terminal, determinate, hyaline. *Conidia* 35–60 × 27–41 µm ($\overset{¯}{x}$ = 46 × 34 µm, n = 50), solitary, acrogenous, ellipsoidal to subglobose, dark brown to black, smooth-walled, dictyospores, with a pore in each cell (Figure 7j), broadly rounded at apex, subtruncate at the base.

*Culture characteristics*: Colonies on PDA reaching 30–40 mm after 4 months at 25℃, circular, with sparse mycelium on the surface, slightly raised, umbonate, greyish-green, and reverse dark brown to black.

*Material examined*: CHINA, Sichuan Province, Chengdu City, Qionglai County, Lugou Bamboo Sea Area, 30°22′37″ N, 103°16′45″ E, 730m elevation, on dead branches of bamboo in freshwater habitat, 12 October 2021, X.D. Yu, A22 (HKAS 124626, holotype); ex-type living culture CGMCC 3.23774, living culture UESTCC 22.0058, UESTCC 22.0059.

*Notes*: The phylogenetic analyses showed that three isolates of *Dematiosporium bambusicola* formed a monophyletic clade in Savoryellaceae and are sister to *D. aquaticum* with absolute statistical support (100% ML/1.00 BYPP). *Dematiosporium Bambusicola* resembles *D. aquaticum* in forming dematiaceous, dictyospores conidia with a pore [26]. However, *D. bambusicola* has larger conidia than that of *D. aquaticus* (35–60 × 27–41 µm vs. 21–28 µm diam) [26]. The conidia of *D. bambusicola* are ellipsoidal to subglobose and dark brown to nearly black, while *D. aquaticus* has globose to subglobose conidia and brown to dark brown [26]. In addition, *D. bambusicola* differs from the latter in having smaller conidiophores (6.5–11.5 × 2–4.5 µm vs. 20–30 × 3.5–4.5 µm) [26]. We hereby introduce the new species based on the distinctiveness of morphology and multi-gene phylogeny.

**Figure 8:**
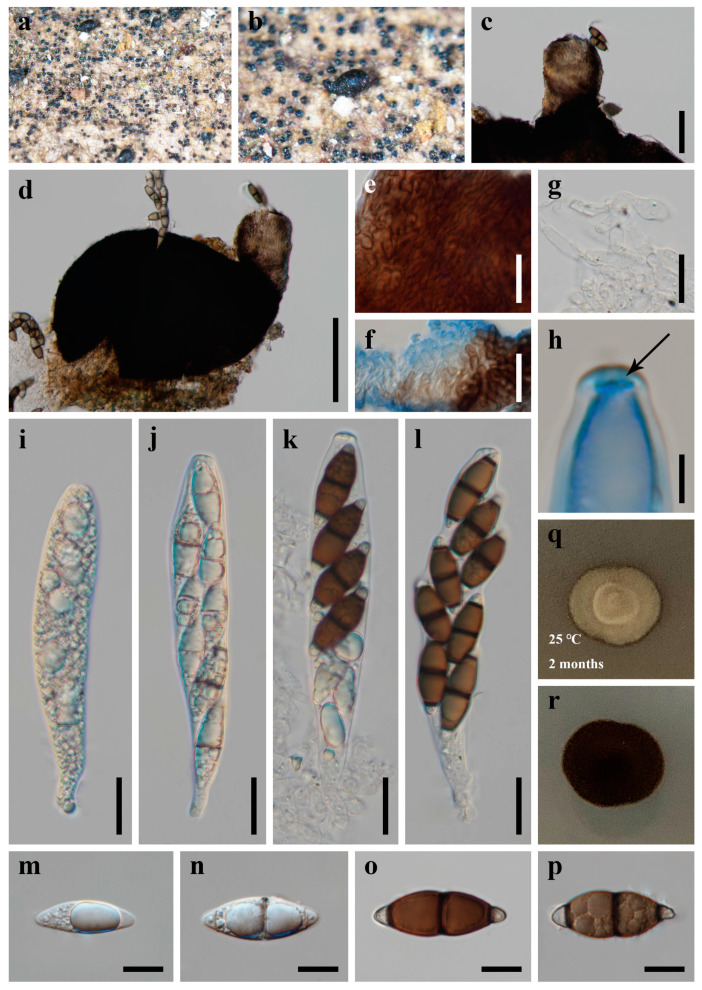
***Savoryella bambusicola*** (HKAS 124627, **holotype**) (**a**,**b**) ascomata on host substrate. (**c**) Neck. (**d**) Squash mounts of ascomata. (**e**,**f**) Peridium. (**g**) Paraphyses. (**h**) Apical ring. (**i**–**l**) Asci. (**m**–**p**) Ascospores. (**q**,**r**) Colonies on PDA, above (**q**) and below (**r**). Scale bars: (**c**) = 50 μm, (**d**) = 100 μm, (**e**–**g**,**i**–**l**) = 20 μm, (**m**–**p**) = 10 μm, (**h**) = 5 μm.

***Savoryella bambusicola*** X.D. Yu, S.N. Zhang, and Jian K. Liu, sp. nov., Figure 8.

*MycoBank*: MB 847554

*Etymology*: Refers to the bamboo host from which the fungi was found.

*Holotype*: HKAS 124627

*Saprobic* on dead bamboo in freshwater habitat. **Sexual morph**: *Ascomata* 270–350 μm high, 180–210 μm diam., scattered or solitary, superficial, black, ellipsoidal, uniloculate, thin-walled, laterally ostiolate, lying horizontal to the host surface with a short, brown neck. *Peridium* comprising several layers of brown, thick-walled cells of *textura epidermoidea. Paraphyses* 4.5–6.5 µm wide, numerous, cylindrical, and somehow swollen, branched, hyaline, septate, constricted at the septa. *Asci* 90–130 × 19–23 µm ($\overset{¯}{x}$ = 119 × 21 µm, n = 30), 8-spored, unitunicate, cylindric-clavate, straight or slightly curved, short pedicellate, rounded at the apex, with an apical truncate non-amyloid apical thickening containing a pore. *Ascospores* 24.5–36.5 × 9–12 µm ($\overset{¯}{x}$ = 30.5 × 10.5 µm, n = 50), uni- or biseriate, straight or slightly curved, fusiform, three-septate, constricted and thickened at the septa, central cells larger and brown, apical cells smaller and hyaline, thin-walled, without sheaths or appendages. **Asexual morph**: Undetermined.

*Culture characteristics*: Colonies on PDA reaching 10–15 mm after 2 months at 25 °C, circular, with dense mycelium on the surface, light gray of the inner ring, and light yellow of the outer ring; in reverse black.

*Material examined*: CHINA, Sichuan Province, Chengdu City, Qionglai County, Lugou Bamboo Sea Area, 30°22′37″ N, 103°16′45″ E, 730m elevation, on dead branches of bamboo in freshwater habitat, 12 October 2021, X.D. Yu, A23 (HKAS 124627, holotype); ex-type living culture CGMCC 3.23775, *ibid*., A20 (HUEST 22.0058, isotype); ex-isotype living culture UESTCC 22.0057.

*Notes*: The phylogenetic result based on SSU, ITS, LSU, *rpb2,* and *tef1α* sequence data showed that the new collections *Savoryella bambusicola* nested in *Savoryella* and formed a distinct lineage (Figure 1). Morphologically, *S. bambusicola* resembles *S. curvispora* and *S. fusiformis* in having eight-spored asci, fusiform, three-septate ascospores [29]. However, they can be recognized as different species; *Savoryella bambusicola* differs from *S. curvispora* and *S. fusiformis* in having relatively broader asci (90–130 × 19–23 vs. 90–115 × 15–17 vs. 80–120 × 9–14) and ascospores (24.5–36.5 × 9–12 vs. 25–28 × 7–10 vs. 25–35 × 6–9.6) [29]. The establishment of the new species *S. bambusicola* is justified by morphological and phylogenetic evidence.

## 4. Discussion

Tibpromma et al. [20] introduced *Canalisporium krabiense* and *C. thailandense* from Thailand. These two species have different conidiogenous cells, but were found to have almost identical ITS and LSU sequences and were phylogenetically clustered together (Figure 1). Koukol and Delgado [47] speculated that DNA cross-contamination happened between *C. krabiense* and *C. thailandense*. Furthermore, they provided synonymy of another species *C. dehongense* under *C. thailandense*. In this study, we deem it as a speculative inference and do not follow this treatment with the following concerns: (1) in Koukol and Delgado [47], the globose to oval conidiogenous cells connected in a chain of *C. dehongense* and *C. thailandense* was also observed in other incorrectly identified *C. caribense*; and inferring from this, they speculated that *C. thailandense* is worldwide and *C. dehongense* could be a further record; (2) the synonymy of *C. dehongense* under *C. thailandense* is only based on morphology; since *C. dehongense* and *C. thailandense* are represented by a single specimen at that time, there was no further evidence to refute the inference [20,46,47]; (3) additional collection of *C. dehongense* is provided in this study (HUEST 22.0057; UESTCC 22.0056), which supported and validated the initial identification of *C. dehongense* in Hyde et al. [46]; (4) Goh and Kuo [48] also pointed out that *C. dehongense* and *C. thailandense* (the basal cell ca. 5µm long vs. 2µm long) are distinct species due to morpho-phylogenetical distinction.

Species of Savoryellaceae have been found on various hosts in aquatic and terrestrial habitats [16], i.e., *Ascotaiwania mauritiana* on *Pandanus palustris* (Pandanaceae) [50], *Ascotaiwania palmicola* on *Iriartea* sp. (Arecaceae) [51], *Savoryella aquatica* on *Machilus* sp. (Lauraceae) [21], *Savoryella lignicola* on *Bambusa* sp. (Poaceae) [21], *Savoryella nypae* on *Nypa fruticans* (Arecaceae) [19], *Savoryella paucispora* on *Pinus* sp. (Pinaceae) [22]. Freshwater species are accommodated in all the genera in Savoryellaceae: *Ascotaiwania* (6 species) [50,52,53,54], *Bactrodesmium* (6 species) [17,55], *Canalisporium* (17 species) [27,28,46,48,56,57,58,59,60], *Dematiosporium* (1 species) [26], *Neoascotaiwania* (4 species) [23,52,61,62], and *Savoryella* (9 species) [16,19,21,29,63]. It seems the members of this family are more likely to be found from monocotyledons and favorable to hard tissue substrates in freshwater habitats.

Asexual morphs have been found in all six genera in Savoryellaceae, of which *Bactrodesmium* and *Dematiosporium* are represented only by asexual morphs; members of *Canalisporium* mostly are asexual morphs with only *C. grenadoideum* having a holomorph; while *Savoryella* is commonly known as a sexual morph with only *S. nypae* and *S. sarushimana* represented by a trichocladium-like asexual morph [19]; *Ascotaiwania* and *Neoascotaiwania* both have holomorphs, but different in asexual morphs (monodictys-like, monotosporella-like, and trichocladium-like vs. bactrodesmium-like) [53,62,64,65,66]. In this study, one sexual morph of *Savoryella* and four asexual morphs of *Canalisporium* and *Dematiosporium* are isolated and identified, which contributed to the taxonomy of Savoryellaceae and the diversity of bambusicolous fungi.

## Figures and Tables

**Figure 6:**
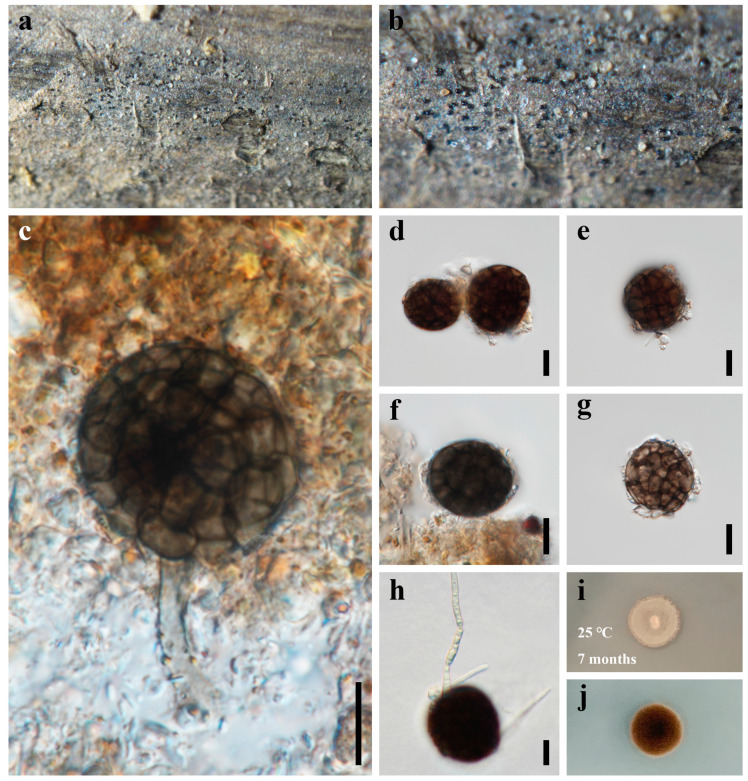
***Dematiosporium aquaticum*** (HUEST 22.0056) (**a**,**b**) colonies on bamboo substrate. (**c**) Conidiophores and conidia. (**d**–**f**) Conidia. (**g**) Fragile conidium. (**h**) Germinating conidium. (**i**,**j**) Colonies on PDA, above (**i**) and below (**j**). Scale bars: (**c**–**h**) = 10 μm.

**Table 1:** Taxa used in the phylogenetic analyses and their GenBank accession numbers. Newly generated sequences are indicated with * and the ex-type strains are in bold. “N/A” sequence is unavailable.

Taxa	Vouchers/Strains/Isolates	GenBank Accession Numbers				
		SSU	ITS	LSU	*rpb2*	*tef1α*
***Ascotaiwania latericolla***	**ICMP 22739**	**N/A**	**MN699390**	**MN699407**	**MN704312**	**N/A**
*Ascotaiwania lignicola*	NIL 00005	HQ446284	HQ446341	HQ446364	HQ446419	HQ446307
*Ascotaiwania lignicola*	NIL 00006	HQ446285	HQ446342	HQ446365	N/A	HQ446308
*Ascotaiwania mitriformis*	HKUCC 3706	N/A	N/A	AF132324	N/A	N/A
*Ascotaiwania sawadae*	SS 00051	HQ446283	HQ446340	HQ446363	HQ446418	HQ446306
*Ascotaiwania uniseptata*	Sloan 5406	N/A	N/A	KT278718	N/A	N/A
*Bactrodesmium abruptum*	CBS 144404	MN699365	MN699391	MN699408	MN704288	MN704313
*Bactrodesmium diversum*	CBS 142448	MN699369	MN699352	MN699412	MN704292	MN704317
*Bactrodesmium leptopus*	CBS 144542	MN699374	MN699388	MN699423	MN704297	MN704321
*Bactrodesmium obovatum*	CBS 144077	MN699375	MN699395	MN699424	MN704298	MN704322
*Bactrodesmium pallidum*	CBS 142449	MN699379	MN699363	MN699428	MN704301	MN704326
*Bactrodesmium spilomeum*	CBS 146104	MN699381	N/A	N/A	MN704303	MN704328
***Canalisporium aquaticium***	**MFLUCC 16-0862**	**MN061353**	**MN061351**	**MN061365**	**N/A**	**N/A**
*Canalisporium caribense*	SS 03839	GQ390253	GQ390283	GQ390268	HQ446421	N/A
Canalisporium dehongense *	UESTCC 22.0056	OQ428250	OQ428266	OQ428258	OQ437183	OQ437176
***Canalisporium dehongense***	**MFLUCC 18-1396**	**MK051035**	**MK051033**	**MK051034**	**N/A**	**N/A**
*Canalisporium elegans*	SS 00523	GQ390255	GQ390285	GQ390270	HQ446423	HQ446310
*Canalisporium exiguum*	SS 00809	GQ390266	GQ390296	GQ390281	HQ446436	N/A
***Canalisporium grenadoideum***	**SS 03615**	**GQ390252**	**GQ390282**	**GQ390267**	**HQ446420**	**HQ446309**
*Canalisporium jinghongense*	SS 03491	GQ390257	GQ390287	GQ390272	HQ446426	HQ446313
*Canalisporium kenyense*	MFLU17-1086	N/A	MH701998	MH701999	N/A	MH708885
***Canalisporium krabiense***	**MFLU 16-1888**	**N/A**	**MH275051**	**MH260283**	**N/A**	**N/A**
***Canalisporium pallidum***	**SS 00498**	**GQ390265**	**GQ390295**	**GQ390280**	**HQ446435**	**HQ446322**
*Canalisporium paulopallidum*	NCYU-106A2-3-1	N/A	MT946658	N/A	N/A	N/A
***Canalisporium paulopallidum***	**NCYU-106A2-3-2**	**N/A**	**MT946659**	**N/A**	**N/A**	**N/A**
*Canalisporium pulchrum*	SS 03773	GQ390263	GQ390293	GQ390278	HQ446432	HQ446319
Canalisporium sichuanense *	**CGMCC 3.23926**	**OQ428254**	**OQ428270**	**OQ428262**	**OQ437186**	**OQ437180**
Canalisporium sichuanense *	UESTCC 22.0060	OQ428255	OQ428271	OQ428263	N/A	N/A
***Canalisporium taiwanense***	**NCYU-108ZQ-D1-1-1**	**N/A**	**MT946663**	**N/A**	**N/A**	**N/A**
*Canalisporium taiwanense*	NCYU-108ZQ-D1-1-2	N/A	MT946664	N/A	N/A	N/A
‘*Canalisporium thailandense*’	MFLU 16-1900	N/A	MH275052	MH260284	N/A	N/A
*Dematiosporium aquaticum*	CBS 144793	MN699385	MN699402	MN699433	MN704307	MN704330
***Dematiosporium aquaticum***	**MFLU 18-1641**	**N/A**	**N/A**	**MK835855**	**MN194029**	**MN200286**
Dematiosporium aquaticum *	UESTCC 22.0055	N/A	N/A	OQ428257	N/A	N/A
***Dematiosporium bambusicola*** *	**CGMCC 3.23774**	**OQ428252**	**OQ428268**	**OQ428260**	**N/A**	**OQ437178**
*Dematiosporium bambusicola* *	UESTCC 22.0058	N/A	OQ428272	OQ428264	N/A	OQ437181
*Dematiosporium bambusicola* *	UESTCC 22.0059	OQ428256	OQ428273	OQ428265	N/A	OQ437182
***Neoascotaiwania fusiformis***	**MFLUCC 15-0621**	**N/A**	**MG388215**	**KX550893**	**KX576871**	**N/A**
*Neoascotaiwania limnetica*	CBS 126576	KT278689	KY853452	KY853513	MN704308	MN704331
***Neoascotaiwania terrestris***	**CBS 142291**	**KY853547**	**KY853454**	**KY853515**	**N/A**	**N/A**
***Pleurotheciella aquatica***	**MFLUCC 17-0464**	**MF399220**	**MF399236**	**MF399253**	**MF401405**	**N/A**
***Pleurotheciella erumpens***	**CBS 142447**	**MN699387**	**MN699406**	**MN699435**	**MN704311**	**MN704334**
*Savoryella appendiculata*	NF 00206	HQ446293	HQ446350	N/A	HQ446442	HQ446327
*Savoryella aquatica*	SS 03801	HQ446292	HQ446349	HQ446372	HQ446441	HQ446326
***Savoryella bambusicola*** *	**CGMCC 3.23775**	**OQ428253**	**OQ428269**	**OQ428261**	**OQ437185**	**OQ437179**
*Savoryella bambusicola* *	UESTCC 22.0057	OQ428251	OQ428267	OQ428259	OQ437184	OQ437177
*Savoryella fusiformis*	SS 00783	HQ446294	HQ446351	N/A	HQ446443	HQ446328
*Savoryella lignicola*	NF 00204	HQ446300	HQ446357	HQ446378	N/A	HQ446334
*Savoryella longispora*	SAT 00320	HQ446301	HQ446358	HQ446379	HQ446449	HQ446335
*Savoryella nypae*	MFLUCC 18-1570	MK543237	MK543219	MK543210	N/A	MK542516
*Savoryella paucispora*	SAT 00867	HQ446304	HQ446361	HQ446382	HQ446452	HQ446338
***Savoryella sarushimana***	**NBRC 105262**	**MK411005**	**N/A**	**MK411004**	**N/A**	**N/A**
*Savoryella* sp.	NF 00205	HQ446305	HQ446362	N/A	N/A	HQ446339
*Savoryella verrucosa*	SS 03331	HQ446298	HQ446355	HQ446376	HQ446447	HQ446332
***Savoryella yunnanensis***	**MFLUCC 18-1395**	**MK411423**	**N/A**	**MK411422**	**N/A**	**MK411424**

## Data Availability

The sequences and alignments data were submitted to GenBank and TreeBASE, respectively.
